# Unsolved severe chronic rhinosinusitis elucidated by extensive *CFTR* genotyping

**DOI:** 10.1002/ccr3.2443

**Published:** 2019-09-27

**Authors:** Fanny Degrugillier, Stéphanie Simon, Abdel Aissat, Natascha Remus, Chadia Mekki, Xavier Decrouy, Aurélie Hatton, Alexandre Hinzpeter, Brice Hoffmann, Isabelle Sermet‐Gaudelus, Isabelle Callebaut, Pascale Fanen, Virginie Prulière‐Escabasse

**Affiliations:** ^1^ INSERM U955 IMRB, Team 5 Créteil France; ^2^ DHU Ageing Thorax Vessel Blood Créteil France; ^3^ Université Paris Est Créteil UPEC Créteil France; ^4^ APHP Centre Hospitalier Universitaire Henri Mondor Laboratoire de Génétique Créteil France; ^5^ Centre Hospitalier Intercommunal de Créteil Centre de Ressources et de Compétences de la Mucoviscidose Créteil France; ^6^ INSERM U955 IMRB, Plateforme Imagerie Créteil France; ^7^ INSERM U1151, INEM Paris France; ^8^ CNRS‐Sorbonne Université UMR7590 Institut de Minéralogie de Physique des Matériaux et de Cosmochimie Paris France; ^9^ APHP Centre Hospitalier Universitaire Necker Centre de Ressources et de Compétences de la Mucoviscidose Service de Pneumo‐Allergologie Pédiatrique Paris France; ^10^ Centre Hospitalier Intercommunal de Créteil Service d'ORL et de Chirurgie Cervico‐Faciale Créteil France

**Keywords:** CFTR, chronic rhinosinusitis, cystic fibrosis, functional studies

## Abstract

Severe chronic rhinosinusitis in children should alert clinicians and extensive *CFTR* genotyping should be performed. We propose that thorough clinical and functional assessment in severe chronic rhinosinusitis is valuable to discover rare mutations which could be treated by CFTR correctors to postpone pulmonary infection.

## Graphical abstract

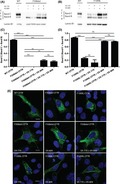

## 1 INTRODUCTION

The European Position Paper (EPOS) clinically defines rhinosinusitis in children by the presence of two or more of the following: symptoms of nasal blockage/obstruction/congestion or nasal discharge; or endoscopic signs of nasal polyps, and/or mucopurulent discharge primarily from the middle meatus, and/or edema/mucosal obstruction; or CT signs of mucosal alteration within the ostiomeatal complex and/or sinuses.^1^ Chronic rhinosinusitis (CRS) in the form of purulent rhinorrhea and cough is very common in the pediatric age‐group. In healthy children, viral infection predominates; when bacteria are involved, *S pneumoniae*, *S aureus*, *S pyogenes* gr. A, *H influenzae,* and *M catarrhalis* are most frequently implicated on culture.^2^, ^3^ In cystic fibrosis (CF), however, the picture is somewhat different. The most frequently identified sinus bacteria are *S aureus* and *H influenzae*, but gram‐negative bacteria such as *P aeruginosa* (*Pa*), *A xylosoxidans,* or *B cepacia* complex are also found.^4^, ^5^, ^6^, ^7^ *Pa* is the most common pathogen in CF and is associated, when chronic infection is established in the lungs, with significant morbidity and mortality. Recent data from Bonestroo et al demonstrated that *Pa*‐positive culture in the upper airways precedes positive lower airway cultures in CF children.^8^ It is therefore essential to detect *Pa*‐positive culture in the upper airways in CF patients.

The US Cystic Fibrosis Foundation (Bethesda) listed diagnostic criteria for CF: one or more of the phenotypic features of the disease with positive immunoreactive trypsin (IRT, a neonatal screening test), positive sweat test, a CF‐causing mutation in each *CFTR* gene, or an abnormal nasal potential difference (NPD).^9^ Most atypical CF patients can be confidently diagnosed with the help of reliable sweat tests and/or genetic analysis. These individuals usually present later in their lives with pancreatic failure and milder respiratory disease. The difficulty occurs when patients present with clinical symptoms suggestive of CF and a sweat chloride value in the intermediate range. Among these subjects, the subset with abnormal NPD or two identified *CFTR* mutations tends to show more severe lung disease.^10^ Similarly, abnormal sweat test or abnormal potential difference across epithelial membranes helps determine risk of developing CF symptoms later in life. However, these functional tests do not predict an individual's clinical syndrome or range of disease severity.^11^

Although carriers of a single CF mutation seem to show higher prevalence of CRS than the general population,^12^ pediatric CRS guidelines and studies of patients with CRS and a single *CFTR* gene mutation do not identify *Pa*‐positive upper‐airway culture as a criterion for going to search for further *CFTR* gene mutations. This may be due to lack of systematic samples of nasal secretions (particularly from the middle meati) in children. However, Raman et al found *Pa‐*positive cultures from the sinuses of p.Phe508del‐heterozygous children with normal or intermediate sweat values (in a group of 58 Caucasian children with chronic CRS, without diagnostic criteria for CF, who underwent sweat testing and genotyping for *CFTR* mutation using an assay that detects 90% of mutations).^13^

Newborn screening detects children having a single *CFTR* mutation escaping from periodic CF centre survey. In fact, the diagnosis of CF cannot be conclusive when the sweat test gives intermediate values and the genetic analysis shows at most one CF‐causing mutation. In the present study, detection of the second F1099L‐CFTR mutation illustrates the importance of a thorough diagnosis for children with severe chronic rhinosinusitis.

## 2 CASE HISTORY

**Figure 1:**
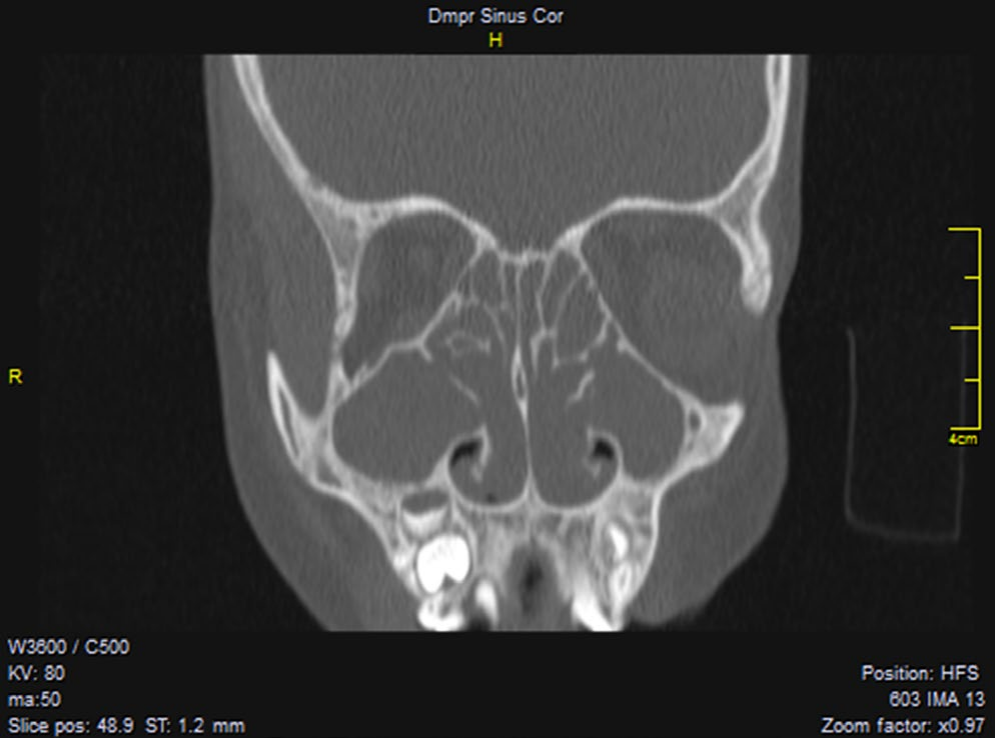
Sinus CT scan. Coronal view of sinus CT scan showing opacities of all sinuses with bilateral medial bulging of the nasal lateral walls

The patient, born in 2004, is a French‐Martinican boy. He had elevated IRT on newborn screening at 69 µg/L (normal < 65 µg/L). A single p.Phe508del mutation was identified and three sweat tests gave negative results, thus excluding CF diagnosis. During childhood, he had dry cough on rare occasions, no gastro‐intestinal symptoms and regular growth for height and weight (in the 90th percentile). At 9 years of age, he was referred to the department of otorhinolaryngology with a history of chronic obstructive rhinosinusitis with mucopurulent discharge since early childhood, olfactory impairment and intermittent facial pain. Prior to referral, the child had received nasal irrigation and local nasal steroids for 12 months, without clinical improvement. Endoscopic examination revealed nasal polyps in both cavities, with medial bulging of the lateral nasal wall and mucopurulent secretion in each middle meatus. Lund‐Kennedy score, measured in each nasal cavity, was up to 12: mucosal edema, presence of discharge and presence of polyps. Protected samples were collected with a suction tube by aspiration from each middle meatus (left and right); *H influenza* was identified. A CT scan showed pan‐sinusitis with total opacification of maxillary, anterior, and posterior ethmoids (Figure 1). Thorough clinical investigation in our CF center showed a FEV1 (forced expiratory volume‐1) of 98%; sputum culture grew *M catarrhalis*, *S aureus,* and *H influenzae*. Chest CT, abdominal ultrasound, and liver function tests were normal. Allergy testing and immunological screening were normal. Lipid‐soluble vitamin levels were low, attributable to low intake rather than to malabsorption, as adequate substitution normalized all values. Sweat chloride level was 24 mmol/L (normal < 30 mmol/L). Extensive genetic testing was performed. Due to the total obstruction of nasal ventilation, bacterial carriage and impaired quality of life, classic image‐guided functional endoscopic surgery was performed, comprising bilateral uncinectomy, anterior ethmoidectomy, and medial antrostomy, leaving significantly enlarged maxillary ostia. During surgery, protected samples collected with a suction tube by aspiration from each maxillary sinus revealed two bacterial strains: *H influenzae* and methicillin‐sensitive *S aureus*. Therefore, amoxicillin and clavulanic acid were prescribed for 15 days and normal saline nasal lavage and topical nasal steroids (mometasone furoate) were continued daily. Samples taken monthly from each middle meatus were negative. Six months later, *Pa* was identified in each middle meatus, and nebulized sonic aerosol therapy with tobramycin (300 mg per nostril, twice daily) was prescribed for 28 days. One month later, samples were negative in each middle meatus. Fifteen months later, the patient presented with recurrence of nasal polyposis, occluding drainage of the maxillary and ethmoidal sinuses. Polypectomy was performed, and protected samples were collected from the maxillary, anterior, and posterior ethmoid sinuses, showing *Pa* infection, although bronchial aspiration during induction anesthesia was negative.

## 3 INVESTIGATIONS

This severe CRS led us to perform extensive *CFTR* genotyping as primary ciliary dyskinesia (PCD) diagnosis was unlikely due to lack of respiratory distress at birth, absence of middle ear involvement and inferior airway infections. Therefore, evaluation of ciliary structure and function to look for PCD were not proposed as first‐line. This extensive *CFTR* genotyping identified c.1521_1523del, p.Phe508del, and an additional mutation in exon 20, c.3297C > A, p.Phe1099Leu. Familial study showed that the patient inherited the p.Phe508del mutation from his father of British origin and the p.Phe1099Leu variant from his mother of Martinican origin. This variant is listed in the dbSNP database (rs747754623) and in the Exome Aggregation Consortium (ExAC) with an allele frequency of 0.05% in the African population (5/10386). It was detected and briefly described in a cohort paper in 2005 in a newborn without symptoms.^14^ In the absence of a follow‐up paper improving the knowledge of the deleterious effect of this mutation and considering the severity of the CRS, we decided to explore expression and function of F1099L in vivo and in vitro.

**Figure 2:**
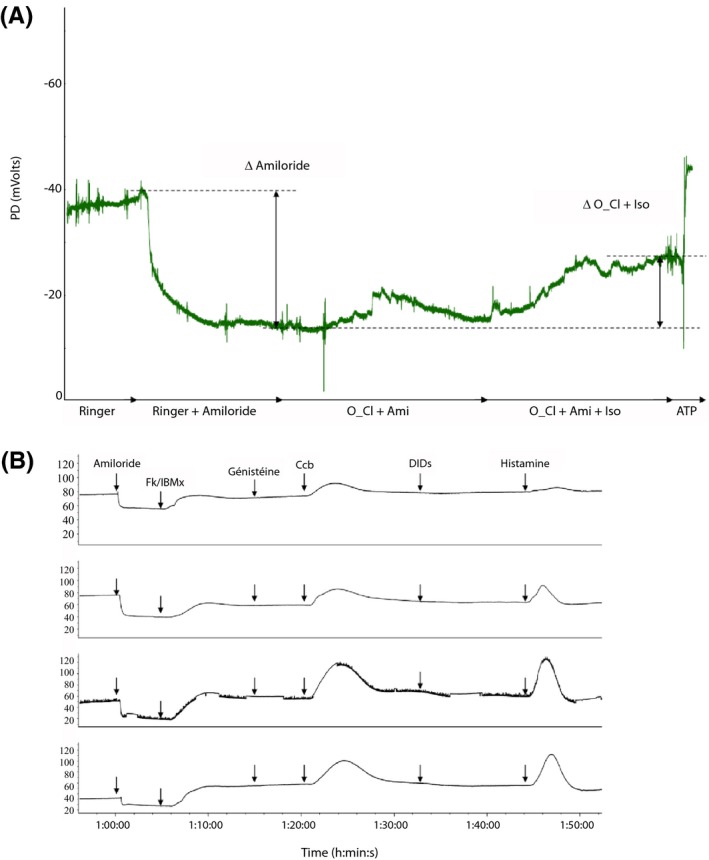
In vivo transepithelial ion transport measurements. Nasal potential difference tracing (A). After the catheter is placed at the point of maximal negative voltage on the nasal mucosa, consistent baseline NPD measurements are measured after perfusion with saline Ringer's solution (S1), and subsequent solutions are perfused in the following order: (S2) 100 µmol/L amiloride, (S3) chloride‐free medium, and finally chloride‐free solution containing 100 µmol/L amiloride and 10 µmol/L isoproterenol (S4). This tracing shows an increased response to amiloride, indicating increased Na^+^ reabsorption, and subnormal total chloride transport, as assessed by the sum of Δ low chloride and Δ isoproterenol. Tracing of short‐circuit current from rectal biopsy tissues (B). After current stabilization, short‐circuit current (Isc) measurements are recorded under the sequential addition of: (1) amiloride (100 µmol/L), followed by (2) cAMP‐induced Cl secretion with 100 µmol/L IBMX + 1 µmol/L forskolin, Fk/IBMX. Cl secretion is further stimulated by adding (3) genistein (30 µmol/L), (4) carbachol, Ccb (100 µmol/L), (5) DIDS (200 µmol/L) is then added to inhibit non‐CFTR Cl channels, followed by reactivation of the CaCC with (6) histamine (500 µmol/L). Selected tracing shows a subnormal cAMP‐dependent and CaCC Cl^−^ secretion, as depicted by the positive deflection following Fk‐IBMX, CCH, and histamine stimulation

Transepithelial ion transport measurements in nasal mucosa showed ENaC channel hyperactivity, as assessed by the high response to amiloride while cAMP‐dependent chloride secretion was normal, as assessed by the response to isoproterenol and low chloride solution (Figure 2A). In the rectal mucosa, forskolin, carbachol, and histamine responses were lower than the usual level, indicating moderate defect in CFTR‐dependent Cl^−^ conduction (Figure 2B). Taken together, these results indicate CFTR dysfunction with residual chloride secretion in this compound heterozygote patient.

**Figure 3:**
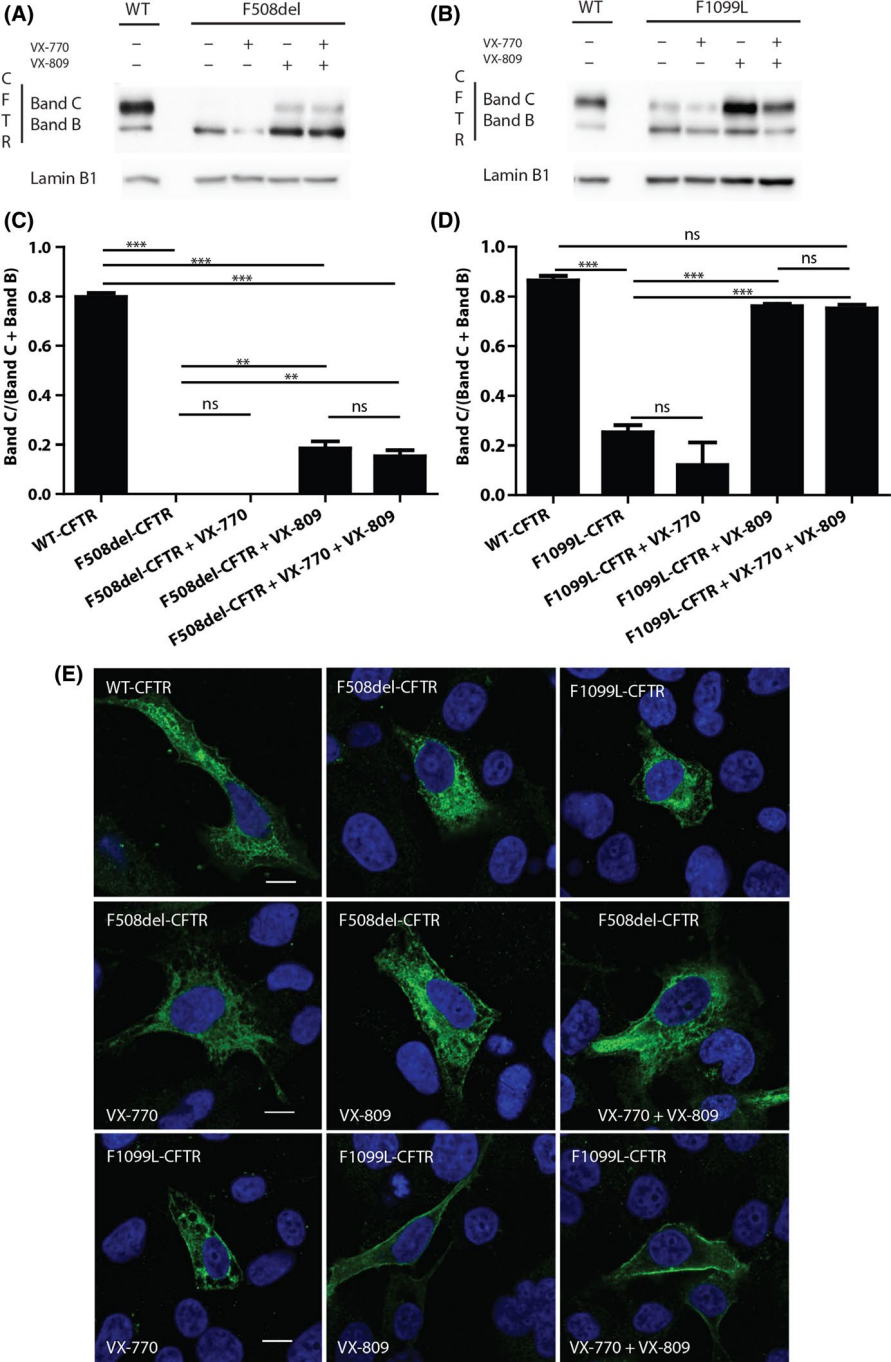
Analysis of WT‐, F508del‐, and F1099L‐CFTR maturation and plasma membrane localization. Representative immunoblot image for F508del‐CFTR (A) and F1099L‐CFTR (B) in untreated and treated HEK cells. Ratio [band C/(band B + band C)] for F508del‐CFTR (C) and F1099L‐CFTR (D). *** indicates a *P*‐value < .001 and ** <.01 on one‐way ANOVA with post hoc Turkey test (n = 3). Representative immunostaining images of cells untreated or treated with VX‐770 or VX‐809 or with both, transfected with vector encoding WT‐, F508del‐, or F1099L‐CFTR (E)

Given these results for in vivo CFTR activity, we decided to investigate the maturation of F1099L‐CFTR protein and the impact of VX‐770 and/or VX‐809 by immunoblot (Figure 3B). By this technique, the mature band of CFTR (band C), which is fully glycosylated, is visualized at higher molecular weight than the immature band (band B). The total extract of transiently transfected HEK293 cells with WT‐CFTR was used as reference, and the ratio of band C to band B plus band C was used as reference value for normal CFTR maturation (Figure 3C). The F508del‐CFTR mutant was analyzed in parallel as control (Figure 3A and C). As expected, WT‐CFTR gave two bands, with band C more intense than band B (Figure 3A and B). For F508del‐CFTR (Figure 3A), in absence of treatment, only band B was visualized by immunoblot. Treatment with VX‐770 alone did not increase the maturation of the mutated protein, as seen by the absence of band C. Treatment with VX‐809 alone or in combination with VX‐770 increased the maturation of the mutant, as seen by the appearance of band C. Quantification and statistical analysis of the ratio C/(B + C) (Figure 3C), indicated that there was no difference in maturation of the mutated F508del‐CFTR in presence nor in absence of VX‐770 alone. A significant difference (*P* < .01) was observed when cells were treated with VX‐809 alone or in combination with VX‐770 in comparison with untreated cells. No difference was detected in cells treated with VX‐809 alone compared to cells treated with VX‐809 in combination with VX‐770. All these findings agreed with the literature. For the F1099L‐CFTR mutant, immunoblot analysis (Figure 3B) revealed a thin band C, indicating that the mutated protein was weakly matured. As F508del‐CFTR, treatment with VX‐770 alone did not increase the intensity of band C for F1099L‐CFTR mutant, whereas treatment with VX‐809 alone or with VX‐809 in combination with VX‐770 clearly enhanced band C. Quantification and statistical analysis of the ratio C/(B + C) (Figure 3D), indicated that there was no significant difference in the maturation of the mutated F1099L‐CFTR in presence nor the absence of VX‐770 alone. A significant difference (*P* < .001) was observed when cells were treated with VX‐809 alone or in combination with VX‐770 compared with untreated cells. No difference was detected in cells treated with VX‐809 alone compared to cells treated with VX‐809 in combination with VX‐770. Moreover, these treatments led to no significant difference in maturation between WT‐CFTR and F1099L‐CFTR mutant.

Taken together, these results showed that F1099L‐CFTR protein matured less fully than WT‐CFTR protein but better than the F508del‐CFTR mutant. Moreover, treatment with VX‐809 alone or in combination with VX‐770 induced complete maturation of F1099L‐CFTR protein.

This decreased F1099L‐CFTR processing and the impact of VX‐770 and VX‐809 drugs were further confirmed by inspection of the subcellular localization of F1099L‐CFTR on confocal microscopy (Figure 3E). As expected, when nontransfected BEAS‐2B cells or cells transfected by empty vector were observed after immunostaining on confocal microscopy, no signal for CFTR was detectable whereas normal sublocalization of WT‐CFTR at the plasma membrane (PM) was observed in cells transiently transfected with the vector encoding WT‐CFTR. In the same conditions, there was no detectable F508del‐CFTR at the PM, while F1099L‐CFTR protein was clearly localized at the PM of the transfected cells. For F508del‐CFTR, as expected, only VX‐809 alone or in combination with VX‐770 led to PM localization of the mutated protein. For F1099L‐CFTR protein, VX‐770 and/or VX‐809 did not affect the detection of the mutated protein at the PM. PM localization was greater in cells treated by VX‐809 alone or combined with VX‐770 than in cells treated by the VX‐770 alone.

These results indicated that F1099L‐CFTR protein was able to be localized at the PM but to a lesser extent than the WT protein. Treatment with VX‐809 alone or in combination with VX‐770 increased this localization, confirming the biochemical and functional data.

**Figure 4:**
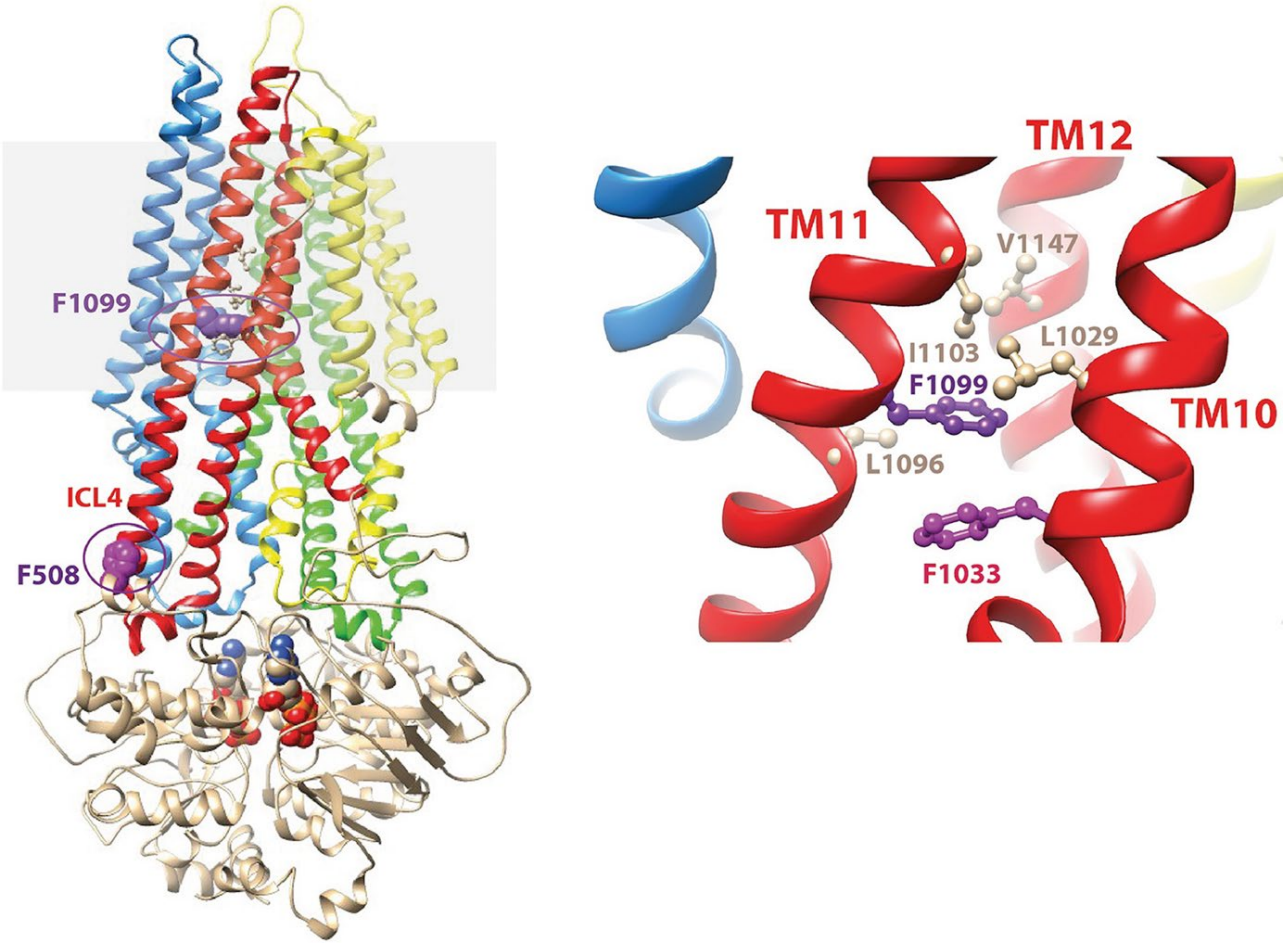
Model of the 3D structure of CFTR. Left: Ribbon representation of the model of the 3D structure of CFTR MSD:NBD assembly in full‐open conformation.^15^ The 4 bundles of transmembrane helices (TM) are colored in red (TM1, TM2, TM3), green (TM4, TM5, TM6), yellow (TM7, TM8, TM9), and red (TM10, TM11, TM12). The two ATP molecules at the interface between the two NBDs are shown, as well as F508 and F1099 (purple circles). The position of the lipid bilayer is shaded gray. Right: Focus on the TM10‐TM11‐TM12 bundle, showing the hydrophobic environment in which F1099 is embedded

Finally, our results are further completed by the 3D structure model (Figure 4). F1099 is located within the membrane‐spanning domains, in transmembrane helix TM11, as predicted from a recent model of CFTR's 3D structure (Figure 4) 15 and contrary to the initial assumption that F1099 was located in intracellular loop 4.^14^ It is in fact buried within the TM10‐TM11‐TM12 bundle, which is one of the four TM bundles forming the nearly rigid bodies of the membrane‐spanning domains, around which conformational changes occur during the gating cycle. The aromatic side‐chain of F1099 strongly interacts with the structure of F1033 (the best aromatic stacking within the model of CFTR's 3D structure), contributing to the stability of the bundle. Mutation of F1099 into a leucine is thus predicted to diminish bundle stability and to impair protein folding. Moreover, the F1099/F1033 couple is located exactly where the upper parts of both TM11 and TM10 bend 20° toward the center of the pore during the gating cycle (transition from open to closed channel conformation).

## 4 DISCUSSION

Currently, in case of chronic rhinosinusitis in children, associating postnasal drip, nasal obstruction and/or purulent discharge, the most frequent diagnosis is allergy. However, when samples from middle meati reveal gram‐negative bacilli such as *P aeruginosa* in a child with a single p.Phe508del mutation, physicians should perform extensive *CFTR* genotyping. This attitude is supported by a recent publication discussing diagnosis in case of rhinosinusitis in children with purulent secretion in each middle meatus.^16^ There is increasing evidence that the sinuses can be the reservoir of bacteria in CF patients. Several studies showed concordance between organisms in upper and lower airways in CF patients, suggesting the hypothesis that the upper airways might influence the patient's pulmonary status.^8^, ^17^ Muhlebach et al 5 reported in 2006 that bacterial concordance in upper and lower airways was more frequent in CF children after 8 years of age. CFTR potentiators and correctors have been developed to correct CFTR protein dysfunction and showed impressive impact on lung function;^18^ these targeted therapies could be implemented in CF children with CRS to postpone pulmonary infection, particularly when *P aeruginosa* is present in the sinuses.

The present study demonstrated that extensive genotyping is essential and productive in patients with atypical presentation such as a single *CFTR* gene mutation and severe chronic rhinosinusitis. Correction of F1099L mutation by VX‐809 offers hope of successful treatment before onset of pulmonary degradation.

## CONFLICT OF INTEREST

All authors declare the absence of conflict.

## AUTHOR CONTRIBUTIONS

FD: involved in realization of the experiences, analysis of the generated data, and writing the manuscript. SS: involved in conception and realization of the experiences, analysis of the generated data, and writing the manuscript. AA: involved in statistical analysis of the generated data. NR: pediatrician involved in clinical follow‐up of the patient. CM: involved in genetic counseling of the family. XD: involved in realization and analysis of confocal experiences. AH: involved in realization of DPN experiences. AH: involved in analysis of experiences. BH: involved in the CFTR‐3D structure analysis. ISG: pediatrician involved in clinical follow‐up of the patient, involved in realization and analysis of DPN experiences, writing the manuscript. IC: involved in analysis of the CFTR‐3D structure and writing the manuscript. PF: is head of the team and involved in supervision of the project, involved in supervision of genetic analysis and writing the manuscript. VPE: ENT surgeon involved in clinical follow‐up of the patient, involved in supervision and coordination of the project and writing the manuscript.
